# Predictive Strategies to Reduce the Risk of Rehospitalization with a Focus on Frail Older Adults: A Narrative Review

**DOI:** 10.3390/epidemiologia4040035

**Published:** 2023-10-08

**Authors:** Rabia Bag Soytas, Elise J. Levinoff, Lee Smith, Alper Doventas, José A. Morais, Nicola Veronese, Pinar Soysal

**Affiliations:** 1 Department of Medicine, Division of Geriatric Medicine, McGill University, Montreal, QC H3G 1A4, Canada; drrabiabag@gmail.com (R.B.S.); elise.levinoff@mcgill.ca (E.J.L.); jose.morais@mcgill.ca (J.A.M.); 2 Center for Health Performance and Wellbeing, Anglia Ruskin University, East Road, Cambridge CB1 1PT, UK; 3 Division of Geriatrics, Department of Internal Medicine, Cerrahpasa Faculty of Medicine, Istanbul University-Cerrahpasa, Istanbul 34320, Turkey; adoventas@yahoo.com; 4 Department of Internal Medicine, Geriatrics Section, University of Palermo, 90133 Palermo, Italy; ilmannato@gmail.com; 5 Department of Geriatric Medicine, Faculty of Medicine, Bezmialem Vakif University, Istanbul 34320, Turkey; dr.pinarsoysal@hotmail.com

**Keywords:** frailty, rehospitalization, older adults, comprehensive geriatric assessment, predictive strategies

## Abstract

Frailty is a geriatric syndrome that has physical, cognitive, psychological, social, and environmental components and is characterized by a decrease in physiological reserves. Frailty is associated with several adverse health outcomes such as an increase in rehospitalization rates, falls, delirium, incontinence, dependency on daily living activities, morbidity, and mortality. Older adults may become frailer with each hospitalization; thus, it is beneficial to develop and implement preventive strategies. The present review aims to highlight the epidemiological importance of frailty in rehospitalization and to compile predictive strategies and related interventions to prevent hospitalizations. Firstly, it is important to identify pre-frail and frail older adults using an instrument with high validity and reliability, which can be a practically applicable screening tool. Comprehensive geriatric assessment-based care is an important strategy known to reduce morbidity, mortality, and rehospitalization in older adults and aims to meet the needs of frail patients with a multidisciplinary approach and intervention that includes physiological, psychological, and social domains. Moreover, effective multimorbidity management, physical activity, nutritional support, preventing cognitive frailty, avoiding polypharmacy and anticholinergic drug burden, immunization, social support, and reducing the caregiver burden are other recommended predictive strategies to prevent post-discharge rehospitalization in frail older adults.

## 1. Introduction 

Frailty is defined as a state of vulnerability resulting from disruptions in organ systems, leading to a reduction in physiological reserves. It is a multidimensional concept that includes physical, cognitive, psychological, social, and environmental factors [1]. Fried et al. described frailty as a syndrome with a biological basis rather than a disease and proposed a clinical fragility phenotype. Accordingly, meeting three out of five phenotypic criteria, including low hand grip strength, low energy, slow walking speed, low physical activity, and unintentional weight loss, is defined as frailty [2]. When frail patients encounter stress factors (illness, trauma, etc.), they are more vulnerable due to the decrease in their reserves, and their adaptation to changing environmental conditions is poor [3]. Therefore, frail older adults often have symptoms such as weakness and fatigue and a reduced tolerance to medical and surgical interventions. Age, gender, immunosenescence (decreased immune system capacity with age, resulting in decreased response to vaccines or infections), under- or overweight, comorbidities, socioeconomic status, low levels of physical activity, cognition, and race are known risk factors for frailty [4]. The biological risk factors for frailty syndrome include age-related inflammatory processes, common chronic diseases, and their interactions with the environment [5,6]. Frailty and pre-frailty appear to be associated with higher oxidative stress and inflammation and lower anti-oxidant parameters; thus, they can lead to physiological decline in multiple body systems, including the skeletal muscle and bone, immune, and cardiorespiratory systems [5,6]. Frailty development also encompasses psychosocial domains such as cognitive frailty, social frailty, depression, and psychological frailty [7,8]. This highlights the need for a more holistic approach to the management of frail patients. 

The prevalence of frailty varies according to both the tool used and the socio-demographic characteristics of the population studied. According to a study conducted in Brazil, the prevalence of frailty in community-dwelling patients was found to be 47.2% [9], while this rate varies between 6 and 86% (median, 42%) in cancer patients [10]. Also, it was shown that while frailty was 6.5% between the ages of 60 and 69, this rate increased to 65% over the age of 90 [11]. In another study conducted on 2816 patients in Turkiye, the frailty prevalence was found to be 10% between the ages of 60 and 69, 31% between the ages of 70 and 79, and 48% for those aged 80 years and above [12]. A systematic review in 2012 reported that when frailty was defined on the basis of physical domains alone, the prevalence was 9.9 percent; when psychosocial aspects were included in the definition, the prevalence increased to 13.6% [13]. The prevalence of frailty in hospitalized patients was detected to be 40% using the criteria of the Cardiovascular Health Study (CHS) [14]. A study with 493,737 participants, 16,538 of whom were frail, showed that patients with frailty are more likely to be female [15].

Since hospitalized older adults are more vulnerable than other individuals of the same age due to their acute medical problems, hospitalization can have a significantly negative impact on frail older adults. Indeed, hospitalization can result in further functional or cognitive decline, as well as emotional distress, in this population. Furthermore, hospital readmission increases medical costs and care burden, and may lead to further deterioration in health status. Therefore, health centers have taken initiatives to reduce costs, such as shortening hospital stays, reducing bed use, and centralizing acute care to optimize resources [16]. Moreover, rehospitalization is known as an indicator of hospital care quality [17]. Predictable readmissions cause a significant burden for patients, caregivers, and healthcare organizations. This highlights the importance of taking necessary and appropriate interventions to reduce predictable hospitalizations. Given this background, the aim of the present review is to highlight the epidemiological importance of frailty in rehospitalization and to compile predictive strategies and related interventions to prevent hospitalizations in frail older adults.

## 2. Reasons for Rehospitalization in Frail Older Adults

Post-discharge rehospitalizations in older adults often lead to a decline in functionality. Importantly, it was suggested that approximately 57% of rehospitalizations are preventable [18]. It is therefore of utmost importance to identify the causes of rehospitalization to inform interventions and practice, especially among those with frailty or at risk of frailty (Table 1).

In a study conducted in Korea investigating the factors affecting readmission in frail older adults, comorbidity and polypharmacy were associated with 30-day readmission, and disease-related factors and grip strength were associated with 90-day and 180-day readmissions, respectively [19]. In a study involving 8071 geriatric patients with a mean age of 83.5 years, which investigated the incidence, diagnosis, and related factors of rehospitalization in Sweden, it was found that a quarter of the patients were rehospitalized within the first 3 months after discharge. The significant risk factors for rehospitalization included age, gender, the number of comorbidities, polypharmacy, and the discharge destination. The most common diagnoses during readmission were heart failure, chronic obstructive pulmonary disease, and pneumonia [20].

The Charlson Comorbidity Index (CCI), which is the most widely used assessment tool to report the presence of comorbid conditions, and the Barthel index (BI), which is used to measure performance in activities of daily living, have both been shown to be directly associated with rehospitalization, prolonged hospital stay, cardiovascular mortality, and mortality in older adults presenting to emergency departments [21]. Moreover, the multimorbidity frailty index, which was created using ICD 10 codes, was found to be a strong indicator for mortality and rehospitalization in frail older adults [22]. These results support that comorbidities and dependence on activities of daily living are risk factors for rehospitalization. Heart failure is one of the most well-known comorbidities associated with rehospitalization. Indeed, greater than 50% of older adults with heart failure experience either rehospitalization or death within one year [23]. Furthermore, the rehospitalization rate was observed to be 61.0% within 1 month in older adults with heart failure [24]. Stroke, hip fracture, chronic obstructive pulmonary disease (COPD), and “poorly controlled diabetes” are other comorbidities known to cause rehospitalization [25]. Recent studies have definitively shown that the presence of a geriatric condition, such as heart failure, falls, a higher number of drugs used, poor overall condition, and functional disability, is a very important reason for rehospitalizations in older adults [26,27]. A strong association was demonstrated between frailty and rehospitalization based on comprehensive geriatric assessment (CGA) parameters [28]. The CGA includes the assessment of older adults for comorbidities, polypharmacy, mental status, mood, nutrition, physical function, social environment, social support, vision and hearing, and the safety of the environment in which they live. One study found that the Multidimensional Prognostic Index (MPI), which is based on the CGA to identify frailty and readmissions within 30 days, predicted rehospitalization among frail older adults [28]. Therefore, it is essential to appropriately define and manage frailty in the outpatient and inpatient clinic setting in order to prevent rehospitalization and its negative consequences.

## 3. The Epidemiological Importance of Frailty for Hospitalization and Rehospitalization in Older Adults

Many studies have shown that frailty is a predictor of adverse health outcomes such as falls, delirium, incontinence, functional dependency in activities of daily living, hospitalization, prolonged hospital stay, rehospitalization after discharge, and mortality [29]. Hospitalization is both a risk factor for frailty and a consequence of frailty [30]. While the prevalence of frailty is 0.8% in community-dwelling older adults [31], it was reported that this prevalence is much higher (81.7%) in acute care units [32]. A hospitalization-associated disability is defined as a decline in the ability of one or more activities of daily living, such as going to the washroom, taking a shower, dressing, transferring from a bed to a chair, or walking independently after hospitalization [33]. Frailty has also been associated with rehospitalization. Although morbidity alone was not associated with rehospitalization, a strong association was found between frailty and rehospitalizations based on comprehensive assessment tools [34]. A prospective cohort study found that the rate of readmissions within 30 days for patients aged 65 years and over was 21.5%, and a readmissions reduction program could decline the rate to 17.8% [35]. In another study, it was observed that in older adults discharged from acute care hospitals, the rehospitalization rates within 1 year were found to be 30% in older adults, excluding those who died during the hospital stay and those who did not have data on rehospitalization [26]. In another study comparing frail and non-frail patients, the rehospitalization rate of frail patients was 2.93 times higher than the rate of non-frail patients [36]. In a study examining the relationship between frailty measured by the Hospital Frailty Risk Score (HFRS) and 30-day rehospitalization in patients with acute myocardial infarction (AMI), heart failure, and pneumonia, frailty was found to be associated with rehospitalization in all three disease groups. In the patient group with AMI, the rate of rehospitalization within 30 days was 10.4% in patients with low HFRS scores (<5), while it was 32.8% in patients with high-risk scores (>15). In patients with heart failure, the rate was 11.8% in patients with low scores and 31.7% in patients with high scores. In patients with pneumonia, the rehospitalization rate was 10.1% in patients with low scores and 26.7% in patients with high scores [37]. Collectively, these findings support that frailty is associated with the rate of rehospitalization, and this rate increases as the degree of frailty increases. Therefore, by identifying the factors that predict frailty and taking the necessary precautions in a timely manner, the prevalence of rehospitalization in older adults can be reduced by lowering the prevalence of frailty.

## 4. Instruments Used to Identify Frailty

It is of utmost importance to develop effective strategies that target the prevention and management of frailty in aging populations and prevent the rehospitalization of patients, since rehospitalization both increases the frailty levels of patients and causes an increase in morbidity and mortality, as well as increases the burden on the health system per se [38]. Therefore, before initiating preventive strategies, it is often recommended to screen older adults for frailty status. However, there is not yet any strong evidence to support the need for routine frailty screening in older adults to improve clinical care and reduce the financial burden on the health system. Nonetheless, there are numerous instruments that are used to identify frailty. Commonly used frailty instruments differ from each other in terms of both the components they contain and their biological basis. This raises the question of which scale is a robust measure of frailty. Therefore, in order to make the correct definition, care should be taken to select an instrument with high validity and reliability, which can be interpreted and practically applicable. The CGA is a detailed holistic approach that evaluates geriatric syndromes in detail and provides physicians with an idea about which areas older adults have problems with so that physicians can help them manage their future care plan [39]. Therefore, although it is the most recommended assessment in the diagnosis of frailty, it is difficult to use the CGA as a screening test in clinical practice because it requires expertise and takes a long time to apply [39,40]. For this reason, there is a need for more efficient screening tools that can be easily applied in clinical practice, are cost-effective, and brief. The physical frailty phenotype [2] and the Frailty Index (FI) [41] are the two most commonly used frailty instruments in clinical practice and research. The frequently used frailty scales are summarized in Table 2. In addition, it was shown that the Mini Nutrition Assessment and Mini Nutrition Assessment-Short Form, which are used in nutritional screening, can also be applied for use in identifying frailty [42,43]. 

There are many factors that affect frailty prevalence, such as the chosen measurement tool, the patient population, and the lack of a uniform standardized test to measure frailty. This has generated the need to identify relevant biomarkers to aid in the diagnosis of frailty [55]. There are metabolic, inflammatory, and hematological biomarkers that have been shown to be associated with frailty. Low-density lipoprotein (LDL) and albumin are metabolic biomarkers that have been shown to be negatively associated with frailty. Tumor necrosis factor-alpha (TNF-α), insulin-like growth factor 1 (IGF-1), and Interleukin (IL) 6 are inflammatory biomarkers known to be positively associated with frailty. The positive relationship between another inflammatory biomarker, C-reactive protein (CRP), and frailty has only been found in hospitalized patients. Hemoglobin is also a hematological biomarker that is negatively associated with frailty [56]. The FT3/FT4 ratio has been found to be associated with frailty [57]. Moreover, Marzetti al. found gender-specific differences among biomarkers among older frail patients [58]. Studies in this area suggest that a single biomarker may not be sufficient for the diagnosis of frailty [59]. Therefore, studies continue to establish biomarker models for frailty [56].

## 5. Predictive and Preventive Strategies

**Figure 1:**
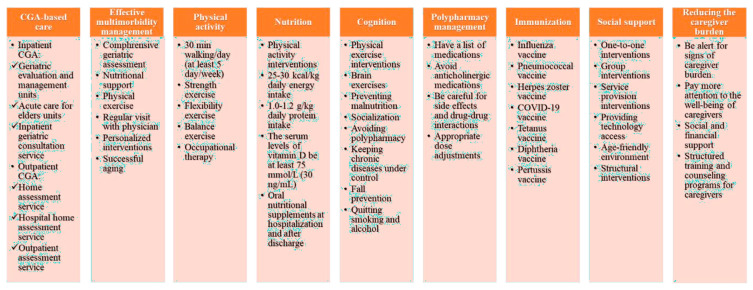
Predictive strategies to investigate frailty syndrome and the likelihood of rehospitalization.

There are some predictive factors identified by studies in the literature for frailty and rehospitalization. Knowing the strategies for prevention from these factors is the most critical point for reducing frailty and therefore rehospitalization (Figure 1).

### 5.1. CGA-Based Care

**Figure 2:**
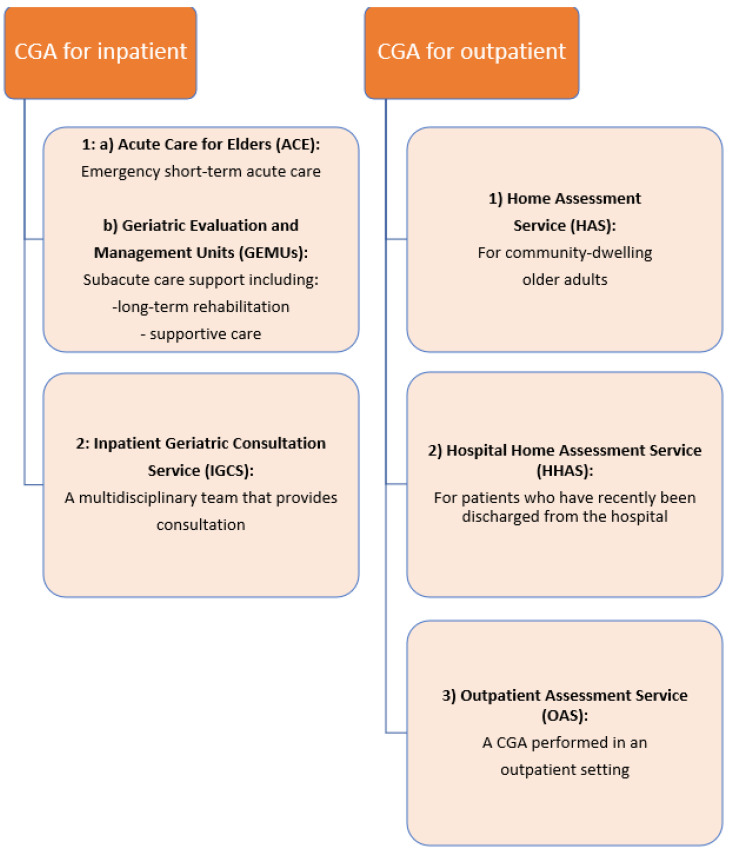
Comprehensive geriatric assessment (CGA)- based care.

The CGA aims to meet the needs of frail patients with a multidisciplinary approach and intervention that includes physiological, psychological, and social domains. This multidisciplinary approach is the first step in creating a care plan for patients, reducing the risk of falls, dependency, hospitalization, and rehospitalization. CGA-based care is important for providing early identification and appropriate management for frail older adults who are the most at risk for complications and adverse health outcomes [60]. There are several different models of CGA-based care, such as acute hospital care, day hospitals, rehabilitation units, nursing homes, and home care services. A meta-analysis in 1993 on different CGA-based care models was seminal for the definition of CGA models for outpatients and inpatients [61]. Accordingly, CGA models for inpatients consist of two types. The first involves geriatric evaluation and management units (GEMUs) and acute care for elders (ACE) units, which include multidisciplinary team recommendations. ACE units include emergency short-term acute care, while GEMUs include subacute care support, which includes longer-term rehabilitation and supportive care. The second type of inpatient CGA is the inpatient geriatric consultation service (IGCS), which consists of a multidisciplinary team that provides consultation advice to doctors who are managing illness in acute hospitalized older adults. The CGA for outpatients can be divided into three subgroups. The first is the home assessment service (HAS) for community-dwelling older adults. The second is the hospital home assessment service (HHAS) for patients who have recently been discharged from the hospital. The third is the outpatient assessment service (OAS), which is a CGA performed in an outpatient setting (Figure 2).

Clinical studies have shown that frail older adults benefit from CGA-based care in an ACE or a GEM unit [62]. In a study involving 151 patients with a mean age of 85.6 years, 64% of whom had an acute functional loss during admission, 93% of patients presented to the emergency department and 67% of patients benefited from CGA [63]. Older adults treated in the CGA unit had a lower 2-year mortality rate than those treated in other care units [62]. It was also found that maintenance in the CGA unit is more cost-effective [64]. Another study examining the relationship of CGA-based care with health-related quality of life (HRQoL) showed that older adults treated in an ACE or GEM unit had fewer reductions in the HRQoL and a lower mortality rate without increased cost [65]. The acute care of frail older adults in the CGA unit was associated with less functional loss and less increased frailty after three months [65]. The CGA-based long-term care plans created at discharge for hospitalized patients are crucial for decreasing readmission rates. The rehospitalization rates were found to be lower at one month after discharge in older adults staying in the CGA-based care unit [66] because the CGA seems to be beneficial in the hospital medical setting for multiple health outcomes, such as reduced delirium and falls, and improves clinical outcomes in oncology, hematology, and in the emergency department [67]. Also, the adequacy of in-hospital treatment and a comprehensive discharge plan was reported to affect the incidence of 30-day readmission in 1263 older inpatients [68].

In light of the above information, CGA-based care is the most important predictive strategy for frailty and rehospitalization, which includes a comprehensive and multidisciplinary approach and has been proven by many studies to reduce morbidity and mortality in older adults.

### 5.2. Effective Multimorbidity Management

Multimorbidity is defined as the presence of two or more chronic conditions and is associated with frailty [69]. The prevalence of multimorbidity is 44–99% in older adults and increases with age [70]. It is known that multimorbidity causes a decrease in the quality of life and an increase in functional dependence and mortality [71,72]. Hospitalization was reported to be two times higher in older adults with multimorbidity [73]. Another study found that multimorbidity increases rehospitalizations [74]. A recent meta-analysis examining the relationship of multimorbidity with hospitalization and rehospitalization in older adults in high-, middle-, and low-income countries found a 2.5-fold positive risk between multimorbidity and hospitalization. It was also shown that multimorbidity increases rehospitalizations by 1.07 times [75]. The multimorbidity frailty index renewed using ICD-10 codes, which is one of the frailty screening tools, was associated with an increased risk of 1-year mortality, unplanned hospitalization, and intensive care admissions, and it was reported that it can be used as a predictor for these risks [22]. Hereby, some of the comorbidities that most commonly cause rehospitalization in frail older adults are listed.

#### 5.2.1. Heart Failure

One of the most important comorbidities, which has been consistently associated with frailty, is heart failure (HF). The guidelines report that frailty, regardless of age, is a multidimensional condition that predisposes patients with heart failure to stress factors and has a poor prognosis [76]. It was found that frailty was high in hospitalized patients with decompensated heart failure, and frailty was associated with age, quality of life, hospitalization, and polypharmacy in these patients [77]. Acute and chronic heart failure diagnosis and treatment guidelines emphasize that frailty should be considered as a part of the evaluation and in the treatment of heart failure patients [78]. In a study comparing the predictive role and diagnostic accuracy of physical (phy-Fi) and multidimensional (m-Fi) frailty scores according to the presence or absence of HF in outpatients over the age of 65, the area under the curve indicated a better diagnostic accuracy with the m-Fi score than with the phy-Fi score for mortality, disability, and hospitalizations, both in the absence and presence of HF. Also, mortality, disability, and hospitalizations were significantly higher in HF patients than in the patients without HF [79]. In a study involving patients with heart failure, in which frailty was determined using the Edmonton Frailty Index, which is a frailty screening tool developed for inpatients, the rehospitalization rates were found to be significantly higher in patients with heart failure at 6 and 12 months after discharge [80]. In addition to age, diabetes, depression, and anxiety have also been found to increase mortality and rehospitalization in patients with heart failure [81,82]. Thus, it is very important to take appropriate interventions to reduce rehospitalization and therefore morbidity and mortality in frail heart failure patients. Cardiac rehabilitation, nutrition, exercise, cognitive and emotional interventional approaches, a reduction in falls, minimizing polypharmacy, and the use of eHealth technology (video cameras that can be monitored by family members, phone applications, or alerting devices that will give an alarm to family members when the patient falls or has syncope) are recommendations given in cardiology guidelines for the management of frail patients [83]. A study conducted to evaluate the multi-domain physical rehabilitation intervention for older adults hospitalized for acute decompensated heart failure (ADHF) showed that this intervention provided an increase in the SPPB score in the third month after discharge. In addition, a negative correlation was found between the SPPB score and rehospitalization. These results show that physical rehabilitation intervention in frail older adults with ADHF increases physical functioning and accordingly decreases rehospitalization [84].

#### 5.2.2. Diabetes Mellitus

Another morbidity known to be associated with frailty is diabetes mellitus [85]. The possible association is likely attributed to insulin resistance or insulin depletion [86]. In older adults with type 2 diabetes, pre-frailty and frailty were reported to increase the risk of mortality, hospitalization, ICU admission, and cardiovascular events, and cause extra health costs [87]. Sarcopenia is the most important target of frailty management in older adults with diabetes since insulin resistance also contributes to the development of sarcopenia by reducing muscle strength and performance. Nutritional support and exercise are important steps in the management of sarcopenia and thus frailty [88]. Frailty was found to be an independent risk factor for non-healing diabetic foot ulcers (DFU) and rehospitalization in older adults with DFU, which is one of the common and important complications of diabetes. Therefore, comprehensive evaluations, personalized interventions, and regular visits with a physician after discharge are recommended for patients who are hospitalized owing to DFU [89].

#### 5.2.3. Chronic Obstructive Pulmonary Disease (COPD)

Another morbidity that causes rehospitalization in older adults is COPD. In a systematic review in 2020 that identified risk factors for rehospitalization following a COPD exacerbation, the rehospitalization rate at 30 days after hospitalization for COPD exacerbation was found to be between 9% and 26%, and at 90 days between 18% and 39% [90]. Comorbidities, previous exacerbations and hospitalizations, and the initial length of hospital stay were found to be the most important factors affecting rehospitalization. The presence of frailty during a COPD exacerbation was associated with readmission and/or mortality [91]. In addition, rehospitalization for COPD was found to be associated with mortality in older adults [92]. A longitudinal study showed that frailty is a predictive factor of readmission within 90 days of hospitalization for acute exacerbations of COPD [93]. In another prospective study examining predictors of 30- and 90-day COPD exacerbation readmissions, previous exacerbations, a higher COPD Assessment Test score at discharge, frailty, a reduced peak inspiratory flow rate (PIFR), and an increased length of stay were found to be associated with 30-day and 90-day readmissions [94]. The results of the study found that patients with more symptoms at discharge (a higher COPD Assessment Test score) had higher rehospitalization rates, showing that discharge assessments have a key role in reducing COPD readmissions.

#### 5.2.4. Dementia

In a systematic review that included 19 articles, the rate of readmission to the hospital was found to be significantly higher in patients with dementia than in patients without dementia [95]. In patients with dementia, the rate of readmissions within 30 days of discharge was 18.91%, and the rate of readmissions within 12 months was found to be two times higher [96,97]. Discharge of the patient before the due time or a poor discharge plan, a lack of post-discharge care services, and frailty of individuals are among the factors that affect readmissions to the hospital in dementia patients [98]. The most common causes of rehospitalization in dementia patients are Behavioral and Psychological Symptoms of Dementia (BPSD), malnutrition, balance disturbances and falls, and frailty [99]. In a study conducted in Taiwan, pneumonia, urinary tract infections, and fall-related fractures were shown to be the most common reasons for hospitalization in dementia patients [100]. Studies show that many hospital readmissions in this population can potentially be reduced or prevented with appropriate interventions. A significant reduction in hospital readmissions within 30 days was observed in the function-focused care and intervention group in the family-centered approach focused on providing support to family caregivers compared to the normal group. In addition, the rate of delirium was lower in the intervention group, and the probability of returning to the previous functional state 2 months after discharge was found to be higher [101]. In another study comparing patients who had and did not have home- and clinic-based palliative care (PC) services in dementia, cancer, chronic obstructive pulmonary disease, and heart failure patient groups, all four disease groups had less hospital use and lower hospital costs in the interventions group [102]. Additionally, while the 30-day readmission rate for people in the Healthy Aging Brain Center Program (HABC) was 11%, this rate was 20% for those in the primary care center [103].

#### 5.2.5. Other Chronic Conditions

End-stage renal disease and non-Hodgkin’s lymphoma are other comorbidities that have been observed to be associated with frailty. Therefore, it is recommended that the CGA be applied regularly to this group of patients in order to detect frailty early and protect older adults from adverse health consequences [40,104]. Furthermore, starting to implement “successful aging” strategies in middle age to prevent the development of these comorbid diseases is an important protective factor.

### 5.3. Physical Activity

There is a large body of work in the literature showing that regular physical activity plays an important role in healthy aging and reduces the risk of chronic diseases and frailty. Importantly, even low levels of physical activity prevent the development of chronic diseases such as type 2 diabetes and cardiovascular diseases [105]. Moreover, physical activity contributes to the improvement in the quality of life by minimizing the dependency of older adults [106]. For this reason, there are various suggestions for older adults to increase their physical activity levels [107].

**Figure 3:**
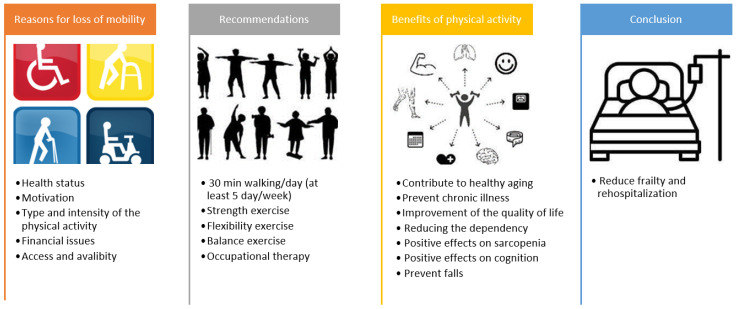
Physical activity as a predictive strategy for frailty and rehospitalization.

Accordingly, it is recommended that patients partake in physical activity at a moderate intensity for at least 30 min a day on at least 5 days a week (Figure 3). In addition, it was shown that physical activity has positive effects on sarcopenia [108] and cognitive functions such as memory, attention, and executive functions [109]. Studies have found that regular and appropriate physical activity (structured exercise) reduces falls [110].

The Lifestyle Interventions and Independence for Elders (LIFE) study was carried out in the USA and based on a combination of walking (target of 150 min/week), strength, flexibility, and balance exercises (Figure 3). The study found that after 2.6 years of follow-up, structured moderate-intensity exercise contributed to the reduction in morbidity and mortality [111].

Importantly, supervised physical activity has been found to be beneficial in hospitalized patients to prevent post-hospital physical and cognitive frailty and rehospitalization [112]. The VIVIFRAIL project is a physical activity promotion program for community-dwelling and hospitalized older adults to prevent frailty and falls [113]. Also, this exercise program has been shown to benefit cognition, muscle function, and mood [114]. In a study involving hospitalized older adults, hospitalization caused a decrease in functional capacity in the control group, whereas this trend was reversed in the VIVIFRAIL group [112]. In addition to physical activity, positive effects of occupational therapy were demonstrated, especially in patients with difficulties in ADLs. A meta-analysis showed that home- and community-based occupational therapy provides moderate improvements in performing ADLs, socialization, and mobility in older adults [115]. Therefore, physical activity advice should be given to frail patients, especially at discharge, in order to reduce rehospitalization rates.

### 5.4. Malnutrition and Sarcopenia

Malnutrition is defined as the deficient or unbalanced intake of energy, protein, and other nutrients that cause adverse effects on tissue/body shape, muscle mass, functionality, and healthy aging [116]. Sarcopenia is a geriatric syndrome that is closely related to frailty and is characterized by low muscle strength, low muscle mass, poor muscle quality, and decreased physical performance [117]. Sarcopenia is considered a component of frailty, and there is considerable overlap between the defining criteria of the frailty phenotype and sarcopenia. The most common criteria applied in frail community-dwelling adults include a slow gait speed (43%) and weakness (54%), which are diagnostic criteria for sarcopenia [118]. For these reasons, maintaining musculoskeletal health is important for the prevention of frailty and associated complications.

Malnutrition, sarcopenia, and frailty are closely related geriatric syndromes, and the presence of one predisposes a person to the occurrence of the other. Malnutrition causes sarcopenia and decreased physical function due to insufficient calorie and protein intake, which, in turn, contributes to frailty. It was reported that sarcopenia and malnutrition risk or malnutrition per se is more common in hospitalized patients and is a predictor of mortality in hospitalized older adults [119]. It is thought that this result is caused by the fact that frail and malnourished older adults living in the community are more likely to be hospitalized. The recently developed Global Leadership Initiative on Malnutrition (GLIM) criteria have been found to have strong prognostic value regarding adverse clinical outcomes and other long-term outcomes in hospitalized patients. Therefore, it is recommended to implement malnutrition screening and methods of prevention in clinical practice, as it is useful both in identifying patients with malnutrition and in predicting possible negative outcomes [120].

An important strategy to minimize malnutrition in hospitalized patients is to reduce the length of hospital stay. In turn, this will reduce the cost on the healthcare system by improving malnutrition management in hospitalized patients. In a study conducted in Holland, it was determined that 74.4% of hospitalized and malnourished elderly patients did not receive enough protein and energy to meet their needs on the fourth day of hospitalization [121]. The recent EFFORT trial by Schuetz et al. demonstrated that individualized nutritional support, including oral nutritional supplements, for hospitalized patients at risk for malnutrition (Nutrition Risk Screening 2002 ≥ 3 points) reduced adverse health effects and mortality within 30 days after admission [122].

Multiple studies have definitively shown that muscle strength, one of the diagnostic criteria for sarcopenia, which is intertwined with malnutrition and frailty, is associated with frailty, hospitalization, rehospitalization, and mortality in older adults. Mortality and rehospitalization were found to be lower in patients with higher hand grip strength in a prospective cohort study [123]. Furthermore, it was observed that patients who had decreased muscle strength during hospitalization had a significant increase in the length of hospital stay and in 3-month and 1-year mortality compared to patients without a decrease in muscle strength [124]. These results emphasize the importance of ensuring the sustainability of the physical performance and the adequate and balanced nutrition of patients during the hospitalization process to maintain muscle strength.

The “Sarcopenia and Physical Frailty in Older People: Multicomponent Treatment Strategies” (SPRINTT) project, which included community-dwelling frail older adults living in Europe, was developed to determine the effect of physical activity and nutrition intervention on preventing mobility disability [125]. In this project, which is stated to be suitable for the target population by all nutritional interventionists, it was determined that it is appropriate to recommend the following for frail older adults: daily energy intake equal to 25–30 kcal/kg body weight (BW), protein intake of at least 1.0–1.2 g/kg BW; and measured serum vitamin D level of at least 75 mmol/L (30 ng/mL). The recently published ESPEN guidelines also recommend daily protein intake amounts of 1.2–1.5 g/kg BW for older adults with acute or chronic illness, and this should even be increased to 2.0 g/kg BW in case of severe illness, injury, or malnutrition [107]. Finally, the ESPEN guidelines suggest that hospitalized older adults with malnutrition or who are at risk of malnutrition should be offered oral nutritional supplements both at hospitalization and after discharge from the hospital to reduce the risk of complications and rehospitalization [126].

### 5.5. Cognition

Studies have shown that frailty increases the risk of cognitive impairment and vice versa [127]. The interaction between frailty and cognitive impairment has led to the term cognitive frailty [128]. Cognitive frailty was defined in a multifactorial manner using different domains of physical, cognitive, psychological, and nutritional scales, of which the Clinical Dementia Rating (CDR) was used to identify older adults with cognitive deficits. Older adults with a CDR of >0.5 who manifest other domains of frailty were classified as cognitively frail [128]. In 2015, Ruan et al. expanded the criteria of cognitive frailty to include “reversible” and “potentially reversible” cognitive frailty. Accordingly, “reversible cognitive frailty” is defined as pre-physical frailty or physical frailty accompanied with pre-mild cognitive impairment (pre-MCI) or subjective cognitive disorder (SCD), and “potentially reversible cognitive frailty” is defined as pre-physical frailty accompanied with mild cognitive impairment (MCI) (Table 3) [129].

The literature has shown that cognitive frailty is associated with increased falls, dependence on activities of daily living, poor quality of life, and increases in hospitalizations and mortality [130]. A study conducted with 3157 community-dwelling older Chinese adults in the USA showed that the rate of hospitalization was approximately 16% in patients with only cognitive impairment and approximately 35% in patients with only physical frailty, while this rate increased to approximately 46% in patients whose frailty included both physical frailty and cognitive impairment [131].

Interventions and the prevention of cognitive frailty are important, as cognitive frailty can be reversible and, if prolonged, can cause increases in morbidity, hospitalization, rehospitalization, and mortality in patients. The literature suggests that physical activity interventions including aerobic training, resistance training, tai chi, and a combination of these can prevent cognitive decline in older adults [132]. Moreover, cognitive training, preventing malnutrition, socialization, avoiding polypharmacy, appropriate chronic disease management, fall prevention, smoking cessation, and alcohol reduction are important interventions that will have positive effects on cognition [133]. All of these may prevent rehospitalization as they can reduce the development of delirium, which often causes admission to the emergency department and hospitalization in frail patients.

### 5.6. Polypharmacy Management

Although there are different definitions of polypharmacy in the literature, the most accepted definition is the use of five or more drugs. Polypharmacy is a geriatric syndrome with an increasing frequency, especially due to the increase in comorbidities with aging, which precipitates multiple prescribers to treat illnesses with medication. As in other geriatric syndromes, polypharmacy causes many adverse health problems such as falls, functional impairments, hospitalizations, increased length of hospital stays, rehospitalizations, and mortality in older adults [134]. Frailty is an adverse health consequence that may be caused by polypharmacy, and frail people have higher rates of polypharmacy [135]. A study found that older adults with polypharmacy have a higher risk of developing frailty at three years compared with those without polypharmacy [136]. In a study conducted by Morandi et al. involving 2735 patients with an average age of 80, they found that those who used seven or more drugs had a four-time higher risk of being rehospitalized within 30 days [137]. Therefore, the awareness and management of polypharmacy in clinical practice are of importance.

The first step in the management of polypharmacy is to have a list of the medications prescribed to the patient, including all vitamins and supplements, and to verify this list with other healthcare professionals. Following this, steps should be made to optimize the effect of drugs and to minimize side effects and drug–drug interactions by reconciling with the patient [138]. It is recommended to consider the Beers, START/STOP, and CRIME methods, which are validated methods used in geriatric populations to evaluate inappropriate drug prescribing [139,140,141]. In addition, it was shown that an evaluation with the CGA is effective in the regulation of appropriate drug therapy, and as a result, this contributes to the reduction in drug-related healthcare costs [142].

Anticholinergic drug burden (ADB) has been found to be associated with falls in frail older adults [143]. In one study, admission to the emergency department and hospitalization were found to be higher in patients with high ADB [144]. Moreover, ADB was associated with high mortality in older adults discharged from acute care hospitals [145]. Therefore, medications with ADB should be avoided, especially in older adults, and if a patient is taking a drug with ADB, a replacement with a non-ADB drug should be considered. Treatment in older adults should be evaluated with a multidisciplinary and multifaceted approach and individualized after CGA. Medications should be prescribed only when necessary, and the profit and loss ratio should be considered. Appropriate dose adjustments should be made, taking into account the patient’s age, comorbidities, and other drugs used.

### 5.7. Immunization

Declines in immune function are the most critical of all the changes that occur with age. It contributes to the increased frequency of infections, malignancies, and autoimmune disorders. Aging affects both the natural and acquired immune system. Macrophage precursors, the first defense mechanism against microorganisms, decrease with age. The lethal functions of macrophages are weakened due to the reduction in nitric oxide and free oxygen radical production. The total neutrophil counts circulating in the bone marrow and blood do not change. However, the phagocytic activities of neutrophils, which are involved in acute inflammation and in the defense against bacterial and fungal microorganisms, are reduced [146]. Dendritic cells are antigen-presenting cells and play critical roles in the formation of antibody responses to antigens. The number of dendritic cells is not affected by age. However, a decrease in plasmacytoid dendritic cell functions is observed. The thymus atrophies is a function of age (thymus involution), causing a significant decrease in the number of naive T cells. B cells, which play a role in humoral immunity, transform into plasma cells, provide an antibody response, and decrease with age, both in their precursors in the bone marrow and in the peripheral circulation. Immunosensence, or the aging of the immune system, does not affect all immune processes equally. One of the conditions most affected by increasing age is the reduced ability of lymphocytes (both B and T cells) to mount effective immune responses after an exposure to new antigens in the form of infections or vaccines [147]. An important concept in immunosenescence is the loss of the precise regulation of inflammatory processes. Older adults show sustained mild cytokine release with a chronic, low-level inflammatory condition that is sometimes referred to as “inflammaging” [148]. This chronic, low-grade inflammatory condition contributes to age-related morbidity, frailty, sarcopenia, disability in activities of daily living, and increased hospitalization rates, hospital stays, rehospitalization, and mortality [149]. A meta-analysis of 35 studies reported that frailty was associated with high inflammatory parameters, particularly IL-6 and CRP [5]. These findings may explain the relationship between inflammaging and frailty [150]. Furthermore, a decreased immune response with aging increases the susceptibility to infections, leading to increased morbidity, frailty, and mortality in older adults. Taken together, immunization is indeed important to reduce vulnerability, hospitalization, and rehospitalization. Influenza, pneumococcal disease, herpes zoster, COVID-19, tetanus, diphtheria, and pertussis are vaccines recommended for older adults [151]. Older adults should be asked about their vaccination status and if there are missing vaccinations, and necessary guidance should be given to complete them. Even if vaccinated, older adults should be informed that the immune response to vaccination is decreased in an advanced age, and they should be encouraged to take the necessary precautions to protect themselves against infection.

### 5.8. Social Isolation–Social Support

Social isolation has been increasing in older adults in recent years, especially in the midst of the COVID-19 pandemic. While physical and cognitive frailty causes social isolation by increasing disability in daily living activities, social isolation is associated with both sarcopenia and physical frailty due to immobility as well as cognitive frailty owing to low brain stimulation [152]. Demographic characteristics such as being older, female gender, a low income and living alone, urinary incontinence, smoking and alcohol abuse, depression and anxiety symptoms, and a low quality of life are known risk factors for social isolation in community-dwelling older adults [153]. Furthermore, studies in the literature have shown that social isolation increases rehospitalization, mortality, and other comorbidities such as malnutrition, cardiac disease, cancer, and falls [154]. In a study investigating social isolation risk factors in hospitalized older adults, it was found that social isolation did not affect mortality but increased the risk of rehospitalization [153]. Therefore, there are some recommendations developed to protect older adults from social isolation [154].

One-to-one intervention is the first of these recommendations, which usually involves voluntary contact with the older adult on a regular basis. This person can be a family member or friend. It was found that regular one-to-one relationships reduce depression and anxiety and have positive effects on health [155]. Group interventions include group therapies that gather individuals around a particular area of interest and aim to increase their social and physical activities. Group interventions were found to be effective in reducing social isolation [156]. Another important intervention is service provision to facilitate older adults’ access to available services. Thus, service provision aims to increase the sustainability of existing services. The development of technology and the increase in the use of social networking sites, the internet, and smartphones have also led to an increase in the quality of life and a decrease in social isolation among older adults. Therefore, providing older adults with technology access and digital literacy may aid in the prevention of social isolation [157]. Moreover, creating an age-friendly environment where older adults live may enable them to live independently and thus aid in the prevention of social isolation [158]. Structural interventions, in which governments encourage the involvement of older people in communities, enable older people to participate in work and social life and may also aid in the prevention of social isolation [138]. Since hospitalization and social isolation both increase frailty, morbidity, and mortality in older adults, it is also important to prevent social isolation in older adults during hospitalization. The Hospital Elder Life Program (HELP) is a multi-component program developed with the aim of preventing functional and cognitive decline in hospitalized older adults [159]. The core intervention protocol of this program includes daily visits, orientation, sleep enhancement, therapeutic activities, vision and hearing adaptation, early mobilization, fluid repletion, and feeding assistance. In addition, geriatric nursing assessment and intervention; geriatric consultation; hand hygiene; aspiration prevention; pain, constipation, and hypoxia management; interdisciplinary rounds; ongoing staff educational programs; post-discharge community links; and telephone follow-ups are other interventions within the program. It was found that HELP, which is widely used in many hospitals around the world, reduces the incidence of delirium and falls, shortens the length of hospital stays, and prevents rehospitalization [160].

### 5.9. Reducing the Caregiver Burden

A caregiver is a person who assumes the burden of care for a person with frailty, disability, or multiple comorbidities. This individual can either live with the person or independently. The caregiver can be a family member, friend, neighbor, or a public or private caregiver who performs this job as a profession. Caregiver burden is defined as the degree to which caregivers feel that their health, psychology, financial situation, and social life are affected by caring for a person [161]. Studies in the literature have shown that female caregivers, those with health problems, low education levels, psychological problems, and more time spent on care have a higher caregiver burden [162].

In addition, since frailty is known to be associated with morbidity, it was reported that caregivers of frail people have more physical, financial, and psychosocial burdens. In a Canadian study, it was shown that care recipients’ physical frailty was associated with subjective caregiver burden [163]. In another study conducted in Turkey, the severity of frailty in care recipients, a low education level in caregivers, and spending more than 8 h per day on care were associated with caregiver burden [164]. If we can reduce the caregiver burden, we can also reduce the development of cognitive frailty, particularly by contributing to the reduction in neuropsychiatric symptoms in dementia patients [165].

Since caregivers undertake a large part of the care of patients during hospitalization, and a hospital environment is a more stressful environment, caring for frail patients who are hospitalized inevitably brings extra burden to caregivers. The caregiver burden affects the caregivers’ physical and psychological health and also affects the care areas, and this may affect the quality of life of the care recipients. Therefore, first of all, both professional healthcare providers and family members should be alert for the signs of caregiver burden and pay more attention to the well-being of caregivers. It is important to increase social and financial support for caregivers, especially during hospitalization. Furthermore, structured training and counseling programs for caregivers may be beneficial in reducing the burden of caregivers.

### 5.10. Palliative Care

Palliative care is an interdisciplinary specialty focused on improving the quality of life for patients with advanced disease and their relatives. Palliative care includes psychological, social, and spiritual care service support and aims to control pain and other symptoms of illness. It also covers the decision-making process regarding medical treatment at the end of one’s life. Palliative care has a regular process such as patient-specific diagnosis, planning, interventions, and follow-up. Hospital palliative care programs have been shown to improve patients’ physical and psychological symptoms and increase caregiver support and family satisfaction [166]. In addition, it was reported that these palliative care programs can reduce hospital and intensive care unit expenditures [167]. In a study conducted in Italy in 2019, in which the relationship between a hospital palliative care unit assessment and hospital outcomes was investigated, the hospital palliative care team found that it reduced the number of hospital deaths, the length of hospital stays, and rehospitalizations [168]. Therefore, applying hospital palliative care programs to frail hospitalized older adults should be considered as one of the measures to reduce rehospitalization, and the programs should be expanded to hospitals all over the world.

## 6. Barriers for Strategies to Reduce Frailty Syndrome and Rehospitalization in Frail Older Adults

There are some limitations and challenges in real-world healthcare settings related to the implementation of strategies developed to prevent hospital readmissions in frail older adults. Some of these challenges relate to identifying and managing frailty in older adults. A lack of a defined protocol for managing frailty, a lack of time to complete the frailty assessment, a lack of consensus on which frailty assessment tool to use, a lack of a multidisciplinary team to support the assessment and management of frailty, the negative response by patients and their families to frailty and its assessment, and patients and their families who do not consider frailty assessment to be important are among the examples of these challenges [169]. There are also some barriers to the implementation of interventions that were shown in the literature to reduce readmissions in frail older adults. For example, the number of geriatricians who can perform CGA is not sufficient in every hospital; age-related physiological changes make it difficult to manage comorbid diseases/multimorbidity; patients and their family members’ non-compliance with interventions; and difficulties in the implementation of physical activity, which is the most important component in the prevention of frailty, due to reasons such as a fear of falling, falling, pain, cognitive problems, or osteoarthritis are some of these barriers. Also, it has become very difficult to reduce social isolation in patients due to the current COVID-19 pandemic. The lack of a sufficient number of palliative care teams in each hospital is another obstacle. All of these barriers prevent the adequate implementation of existing measures. Therefore, there is a need for studies to investigate what can be done to identify and remove these barriers to increase the applicability of predictive strategies.

## 7. Conclusions

Rehospitalization, which is one of the consequences of frailty, negatively affects the quality of life of both older adults and caregivers and causes adverse health problems in older adults. Rehospitalization is an indicator of hospital care quality, and the unplanned rehospitalization of older adults poses significant health, financial, and social problems. Therefore, it is very important to screen, define, and manage frailty correctly in order to reduce the rehospitalization risk. Physical activity, malnutrition, cognition, polypharmacy, immunization, social isolation, and caregiver burden are factors known to increase frailty and rehospitalization in older adults. Knowing the predictive factors and creating strategies to prevent frailty are necessary in order to prevent both rehospitalization and its negative consequences such as worsening quality of life, increased dependence, morbidity, and mortality.

## Figures and Tables

**Table 1:** The causes of rehospitalization in older adults.

1. **Patient-related causes:** ***(a)*** ***Socio-demographic causes:*** Age Female gender Low level of education Living situation (nursing home resident, rurality, etc.) ***(b)*** ***Health-related causes:*** Malnutrition Reduced mobility Cognitive impairment Heart failure COPD Diabetes Hypertension Kidney failure Cerebrovascular disease Atrial fibrillation Depression Presence of cancer with or without metastasis Disability Polymorbidity Polypharmacy Pressure ulcers Anemia Falls Infections (pneumonia or urinary tract infection) Drug side effect Electrolyte imbalances (such as hyponatremia)	2. **Previous hospitalization causes:** Number of admissions within 1 year (≥4) Prolonged hospital stay during the last hospitalization Emergency or ICU admission during the last hospitalization Diagnosis in the prior hospitalization, which is one of the factors that increase the risk of rehospitalization Not applying the necessary interventions in the previous hospitalization Failure to provide adequate information to patients and their family members at discharge Prescribing medications that increase the risk of rehospitalization due to side effects at discharge Failure to perform follow-up regularly after discharge

Abbreviations: COPD: chronic obstructive pulmonary disease.

**Table 2:** Frequently used frailty scales.

Frailty Instrument	Domains İncluded						Scoring	Time to Complete	Setting
	Physical Function	Physical Activity	Cognition	Comorbidity	Weight Loss	Other (Social, Demographic, etc.)			
**Physical Frailty Phenotype [2]**	yes	yes	no	no	yes	no	Frailty: ≥3 items Pre-frailty: 1–2 items Robust: 0 items	5–10 min	- Primary care - Hospital - Long-term care home
**Frailty Index [41]**	yes	no	yes	yes	no	yes	Suggested cutoff score for frailty: >0.5	Varies depending on number and type of measures included	- Primary care - Hospital - Long-term care home
**Electronic Frailty Index [44]**	yes	no	yes	yes	yes	yes	Severe frailty: score >0.36 Frailty: score >0.24–0.36 Mild frailty: score > 0.12–0.24 Fit: score ≤ 0.12	Varies depending on number and type of measures included	- Primary care
**FRAIL Scale [45]**	yes	no	no	yes	yes	no	Score range 0 to 5 No frailty = 0 deficits Intermediate frailty = 1 or 2 deficits Frailty = 3 or more deficits	Less than 5 min	- Primary care - Hospital - Long-term care home
**Clinical Frailty Scale [46]**	yes	yes	no	yes	no	yes	Frailty: score ≥5	Less than 5 min	- Primary care - Hospital - Long-term care home
**Edmonton Frail Scale (EFS) [47]**	yes	yes	yes	yes	yes	yes	Frailty: score ≥ 7	5–10 min	- Hospital
**Study of Osteoporotic Fracture (SOF) Frailty Measure [48]**	yes	yes	no	no	yes	no	Frailty: ≥2 items Pre-frailty: 1 item Robust: 0 items	Less than 5 min	- Primary care - Hospital
**Geriatric 8 Frailty Questionnaire for Oncology (G8 ) [49]**	yes	no	yes	no	yes	yes	Frailty: score ≤ 14	Less than 5 min	- Primary care - Hospital - Long-term care home
**Vulnerable Elders Survey (VES-13) [50]**	yes	yes	no	no	no	yes	Frailty: score ≥ 3	Less than 5 min	- Primary care
**Short Physical Performance Battery [51]**	yes	yes	no	no	no	no	Frailty: score ≤ 9	5–10 min	- Primary care
**Multidimensional Prognostic Index (MPI) [52]**	yes	no	yes	yes	yes	yes	Frailty: Low risk: 0 Moderate risk: 0.5 Severe risk: 1	varies depending on number and type of measures included.	- Hospital
**Frailty Risk Score [53]**	yes	yes	no	yes	no	yes	Very good: score < 45 Good: score 45–50 Moderate: score 51–55 Poor: score 56–61 Very poor: score > 61	5–10 min	- Hospital
**Hospital Frailty Risk Score [54]**	yes	no	yes	yes	no	no	Low risk: score < 5 Intermediate risk: score 5–15 High risk: score > 15	5–10 min	- Hospital

**Table 3:** Definitions of cognitive frailty types.

	Pre-Physical Frailty	Physical Frailty
**Pre-MCI/SCD**	Reversible cognitive frailty	
**MCI**	Potentially reversible cognitive frailty	Cognitive frailty

Abbreviations: MCI: Mild Cognitive Impairment; SCD: Subjective Cognitive Disorder.

## Data Availability

No new data were created or analyzed in this study. Data sharing is not applicable to this article.
